# Comparing 2-nt 3' overhangs against blunt-ended siRNAs: a systems biology based study

**DOI:** 10.1186/1471-2164-10-S1-S17

**Published:** 2009-07-07

**Authors:** Preetam Ghosh, Robert Dullea, James E Fischer, Tom G Turi, Ronald W Sarver, Chaoyang Zhang, Kalyan Basu, Sajal K Das, Bradley W Poland

**Affiliations:** 1 School of Computing, The University of Southern Mississippi, 118 College Drive, Hattiesburg, MS-39406, USA; 2 EMS Computational Sciences, Pfizer Inc., PGRD-Groton Laboratories, Eastern Point Road, Groton, CT-06340, USA; 3 EMS Protein Cell and Assay Technology, Pfizer Inc., PGRD-Groton Laboratories, Eastern Point Road, Groton, CT-06340, USA; 4 Department of Computer Science & Engineering, The University of Texas at Arlington. 416 Yates St., Arlington, TX-76019, USA; 5 Covance Central Labs, 8211 Scicor Dr, Indianapolis, IN-46214, USA

## Abstract

In this study, we formulate a computational reaction model following a chemical kinetic theory approach to predict the binding rate constant for the siRNA-RISC complex formation reaction. The model allowed us to study the potency difference between 2-nt 3' overhangs against blunt-ended siRNA molecules in an RNA interference (RNAi) system. The rate constant predicted by this model was fed into a stochastic simulation of the RNAi system (using the Gillespie stochastic simulator) to study the overall potency effect. We observed that the stochasticity in the transcription/translation machinery has no observable effects in the RNAi pathway. Sustained gene silencing using siRNAs can be achieved only if there is a way to replenish the dsRNA molecules in the cell. Initial findings show about 1.5 times more blunt-ended molecules will be required to keep the mRNA at the same reduced level compared to the 2-nt overhang siRNAs. However, the mRNA levels jump back to saturation after a longer time when blunt-ended siRNAs are used. We found that the siRNA-RISC complex formation reaction rate was 2 times slower when blunt-ended molecules were used pointing to the fact that the presence of the 2-nt overhangs has a greater effect on the reaction in which the bound RISC complex cleaves the mRNA.

## Introduction

RNA interference (RNAi) refers to a post-transcriptional gene silencing mechanism with potential therapeutic application for the treatment of various diseases including cancer, viral infections, and neurodegenerative disorders [1]. The RNAi pathway involves the introduction of a small interfering double stranded (ds) RNA, siRNA, promoting degradation of a target mRNA [2]. RNAi molecules have sequence complementation to that of the siRNA antisense (or guide) strand ultimately inhibiting the translation of the encoded protein. Currently, RNAi is being extensively used to study the functions of individual genes based on its intrinsic property of regulating the expression of a distinct mRNA species in mammalian cells [3,4] as well as offering additional improvements over alternative technologies including knock-out by homologous recombination and antisense. In Ref. [5], the authors performed a comparative analysis of the suppressive effects of three knockdown methods, namely, methods based on RNA interference (RNAi), antisense ODNs, and ribozymes, using a luciferase reporter system. Their dose-response experiments revealed that the IC_50 _value for the siRNA was about 100-fold lower than that of the antisense ODN besides providing useful information about the positional effects in RNAi.

An important consideration towards selecting an effective siRNA-based gene silencing tool is the duration of effect and efficacy of a candidate molecule. Extensive studies determining the intensity of gene silencing mediated by siRNA were reported in Ref. [6] &[7] by finding the optimal sequence of siRNA. Upon introduction of siRNAs with an optimized sequence into cells, the target mRNA is degraded resulting in a lowering of the corresponding protein. It has been reported that 21-nt siRNAs with 2-nt 3' overhangs are more potent than other types of siRNA molecules in terms of reduction in protein levels [8]. Recent studies have reported a higher potency effect due to 27-mer RNA duplexes which we will consider in the **Discussions **section.

Upon introduction into a cell, a siRNA molecule will be diluted over time due to its degradation and cellular proliferation resulting in a decrease in its effective concentration. Consequently, the expression level of the target gene will return to a normal level after the gene silencing period, dependent upon the number of siRNA molecules actually entering the cell. To use siRNA for silencing target gene expression, it is important to understand how long the target mRNA or protein is suppressed by the siRNA. Maximal inhibitory efficiency of siRNA, a parameter that has frequently been used to express the potency of each siRNA, should be discussed in context with the duration or persistence of its effect.

**Figure 1:**
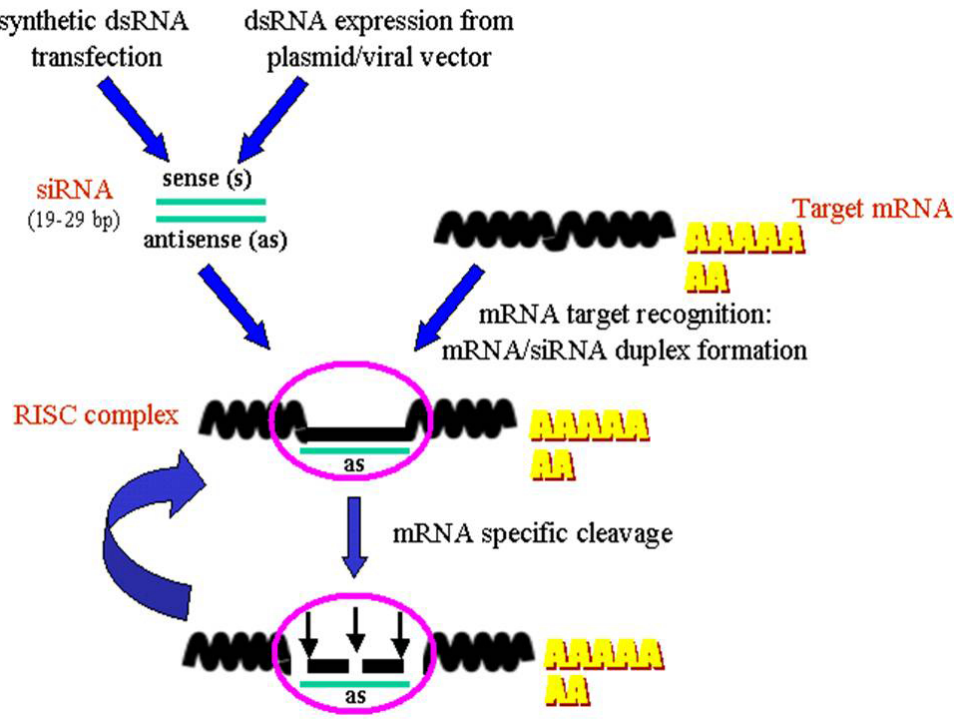
**RNAi system overview**.

Figure 1 illustrates the RNAi system which consists of the following major steps [9]. Long dsRNAs (e.g. >200 base pairs) get processed into 19–25 nucleotide (nt) small interfering RNAs (siRNAs) by an RNase III-like enzyme called Dicer (initiation step) or in the case of mammalian RNAi are introduced to cells directly as "active"(19–25 nt) siRNA molecules. The siRNAs assemble into endoribonuclease containing complexes known as RNA-induced silencing complexes (RISCs), unwinding in the process. Activated RISC then binds to complementary transcript by base pairing interactions between the siRNA antisense strand and the mRNA. The bound mRNA is cleaved and sequence specific degradation of mRNA results in gene silencing. We refer to the aberrant pieces of RNA after cleavage as "garbage RNA" (referred to as gRNA in this paper). It has been reported that a substantial fraction of the siRNAs in *Caenorhabditis elegans *is not derived directly from the introduced dsRNA [10]. To explain this, two amplification routes have been proposed: primed and unprimed amplification [11-13]. In both cases, RNA-dependent RNA polymerase (RDR) synthesizes dsRNA: in the case of primed amplification siRNA binds to mRNA to initiate dsRNA synthesis, whereas in the case of unprimed amplification the mere presence of aberrant garbage RNA is sufficient to trigger RDR. In short, the generally accepted pathway of RNA silencing consists of the degradation of mRNA via RISC and an amplification pathway through RDR.

In this study, we use computational modeling to investigate the potency effects of the widely used 21-nt siRNAs with 2-nt 3' overhangs as compared to blunt-ended siRNA molecules to assess the effectiveness of the latter as an alternative structural entity. We have developed a simple systems biology simulation to quantitatively assess both the intensity and duration of gene silencing by siRNA. The stochastic simulation framework presented here allows us to predict some quantitative and qualitative aspects of the RNAi system for the two different types of siRNAs.

## Materials and methods

The reaction model was built to investigate the potency difference between 2-nt 3' overhangs and blunt-ended siRNA molecules. We identified that the siRNA-RISC complex formation reaction rate was altered due to the different siRNA molecular structures. We assume that once the bound RISC complex is formed, it will cleave the mRNA with the same rate irrespective of the presence of the overhangs on the siRNA. Findings in Ref [14] suggest that the direction of Dicer processing confers some kind of functional polarity, possibly at the level of RISC loading. However, the mRNA cleavage reaction rate has not been explicitly studied for different types of siRNAs as yet.

Hence, our first contribution is a chemical kinetic theory based analytical model for measuring the rate constant of the siRNA-RISC complex formation reaction. The two different rate constants predicted by this model for the different siRNA structures were then fed into a stochastic simulation (using the widely used Gillespie stochastic simulator [15]) to study the potency effects. This gave us a relative quantification between the gene silencing period and the concentration of the two types of siRNA molecules required to keep the mRNA count at the same level. We also incorporated the gene transcription/translation reactions to study whether a burst in mRNA production [16] has a role to play in the RNAi system.

### Reaction model

Consider the elementary reaction with three types of molecules siRNA, RISC and the siRNA-RISC complex:



We divide the reaction event into two independent micro-events as follows; 1) Random collisions between the reactants; this allows us to compute the probability of collision (p_c_) between the reactant molecules. 2) A reaction will occur only when the kinetic energy of the colliding reactant exceeds the activation energy requirement for the reaction. Using these two events, allows us to compute the probability of reaction (p_r_).

The total probability for reaction after a collision is hence the joint probability of these two events. To model this reaction analytically in the time domain, we first assume that the siRNA molecules enter the cell one at a time. Note that siRNAs are typically delivered via transfection thereby introducing a bolus of molecules into the cell. Thus, we need to consider the effective number of siRNAs in the cell while computing the binding rate. We will show how the computations change while we consider a certain concentration of siRNA molecules for deriving the overall binding rate subsequently in this section. We also assume that the cell contains a fixed number, n_2_, of RISC molecules. Note that while modeling the reaction, it is not necessary to consider the fact that the siRNAs or RISC complexes can also take part in other reactions with other reactants or can degrade independently. The time domain model is based solely on the current instance of these two reactants in the cell as the time taken to complete this reaction will generally be less in comparison to the time taken to degrade a siRNA or a RISC molecule. The idea here is to discretize this reaction from other competing reactions that can change the concentrations of the siRNA/RISC molecules. Though this approximation might lead to less accurate predictions of the binding rate for this reaction, we can still consider the effects of such competing reactions by the system simulation of the RNAi pathway (as shown later).

**Figure 2:**
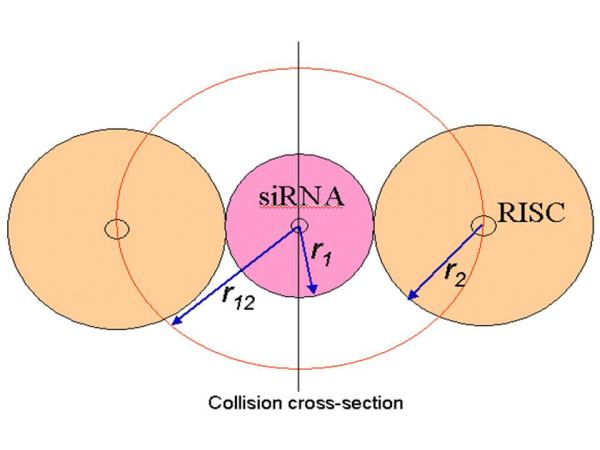
**Schematic diagram of siRNA and RISC molecules**.

**Figure 3:**
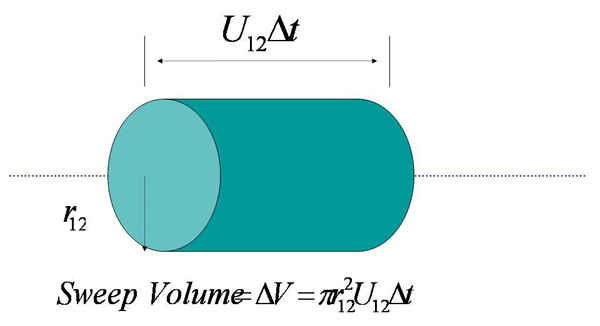
**Volume swept out by the siRNA molecule in time Δt**.

We follow the principles of collision theory for hard spheres [17] to model the chemical kinetics of the reaction. As shown in Figure 2, we model each reactant molecule as a rigid sphere with radii r_1 _and r_2 _for the siRNA and RISC molecules respectively. We define our coordinate system such that the RISC molecule is stationary with respect to the siRNA molecule for the reaction, so that the siRNA moves towards the RISC molecule with a relative velocity U_12_. The siRNA molecule moves through the cell cytoplasm to sweep out a collision cross section A = *π *r_12_^2 ^(as illustrated in Figure 3), where r_12 _is the collision radius given by: r_12 _= r_1 _+ r_2_. If the center of a RISC molecule comes within a distance of r_12 _of the center of a siRNA molecule, they will collide which might result in a successful reaction.

To discretize the system, we consider the dynamics of this process within a small time Δt. We assume that the temporal reaction process is an independent sequence of events separated by Δt. In time Δt, the siRNA molecule sweeps out a volume ΔV given by: ΔV = *π *r_12_^2 ^U_12 _Δt. Note that, in Figure 3 the siRNA molecule actually sweeps out a cylindrical volume that allows us to estimate ΔV as the length of the cylinder in time Δt, thus is given by U_12 _Δt.

Now, the probability of a siRNA molecule being present in the collision volume ΔV is p_siRNA _= 1. This is because we have already assumed that one siRNA molecule entered the cell creating a collision volume of ΔV.

Probability of at least one molecule of RISC being present in an arbitrary uniformly distributed ΔV in V is p_RISC _= ΔV.n_2_/V, where V denotes the cell volume. Ideally V should be the volume of the cytoplasm, which can be approximated by the entire cell volume. The probability that a siRNA molecule collides with a RISC molecule in Δt is given by:

(1)

Thus we have a stochastic sequence of events characterized by the probability of collision, and it is important to determine whether the collision will create the reaction. To complete the reaction, the molecules have to bind to each other. Different types of bonds (ionic, covalent, hydrogen etc.) require different activation energies for binding. We next assume that the colliding molecules must cross an energy threshold, defined by the free energy E_Act_, to provide the energy to react. Also, we assume that only the kinetic energy directed along the line of centers of the two reacting molecules contribute to the reaction as the effects of other forces (e.g. coulomb force) can been captured by the velocity distribution of the siRNA molecules in the cell.

These two assumptions define the probability of another independent event: successful reaction after collision denoted by p_r_. The kinetic energy of approach of a siRNA towards the RISC molecule with relative velocity U_12 _is E = m_12_.U_12_^2^/2, where m_12 _= m_1_.m_2_/(m_1_+ m_2_) = the reduced mass, m_1 _= mass (in gm) of a siRNA molecule and m_2 _= mass (in gm) of a RISC molecule. We also assume that as E increases above E_Act_, the number of collisions that result in reaction also increases linearly. Thus the probability for a reaction to occur, p_r_, is given by:

(2)

and hence, the joint probability, p, for collision and reaction is given by:

(3)

Until now we were working with a fixed relative velocity U_12 _for the siRNA molecules. The velocity distribution of the macromolecules inside a cell capturing the effects of the different forces as obtained from Molecular Dynamic Simulation is generally found to be comparable to the Maxwell-Boltzmann distribution [18,19]. Hence, to approximate the relative velocity of the siRNA molecules, we use the Maxwell-Boltzmann distribution of molecular velocities for a species of mass m given by:

(4)

where k_b _= Boltzmann's constant = 1.381 × 10^-23 ^kg-m^2^/s^2^/K/molecule and T is the absolute temperature at which the reaction occurs. Replacing m with the reduced mass m_12 _of the molecules, we get,

(5)

The term on the left hand side of the above equation denotes the fraction of siRNA molecules with relative velocities between U and (U+dU). Summing up the collisions for the siRNA molecules for all velocities we get the probability of reaction, p, as a function of temperature only as follows:

(6)

Now, recalling E = m_12_.U_12_^2^/2, i.e., dE = m_12_U_12_dU and substituting into the expression for f(U, T)dU, we get:

(7)

Thus we get:

(8)

To consider a certain concentration of siRNA molecules, we assume that n_1 _number of siRNA molecules are present in the cell. This will increase the probability of a successful reaction by a factor of n_1_, and hence we have:

(9)

We discretize the temporal reaction process as a Bernoulli trial process. Next we compute the average time taken to complete the reaction with this probability. Let us assume that the molecule composition does not change during the reaction time. This is valid due to the very short time for reaction compared to the time taken for a potential change in the reaction environment for the associated molecules. The molecules try to react through repeated collisions. If the first collision fails to produce a reaction, they collide again after Δt time units and so on. We can interpret p as the probability of a successful reaction in time Δt. Thus the average time of reaction, T_avg_, can be approximated by summing up the times taken for a successful reaction by the first collision, or that by the second collision and so on. Thus we get:

(10)

Similarly, the corresponding second moment, T_2ndmoment_, can be formalized by

(11)

This gives us the first and second moments of the reaction time, which is considered to be a random variable. The binding rate for this reaction can be easily estimated as 1/T_avg _although the reaction is essentially stochastic. Note that, the expression for the binding rate from our model is exactly the same as that derived by chemical kinetic modeling of reaction rates. However, our model allows us to study the inherent stochasticity of the reaction considered.

### Estimating the reaction rate constant for the siRNA molecules

The above model was used to study the binding rate of siRNA molecules. Note that the model requires an estimate of the activation energy for the siRNA-RISC complex formation reaction. However, it is very difficult to experimentally measure the activation energy required for a reaction. The activation energy is generally derived from the heat of reaction (-ΔH^0^) by using the Polyani equation [17]. But it is equally difficult to measure the heat of reaction specifically for those occurring inside the cell.

We observed that the siRNA-RISC reaction occurs when the siRNA duplex breaks down into two single-stranded molecules one of which enters the reaction (the antisense strand), while the other (the sense strand) disintegrates. The antisense strand enters into a docking reaction with the RISC complex. Thus, we can rewrite the siRNA-RISC complex formation reaction in the following form:



Note that we treat the double stranded siRNA as a complex between the sense (siRNA_sense_) and antisense (siRNA_antisense_) strands. Referring to the reaction model discussed above, the two colliding macromolecules will be the siRNA_sense _siRNA_antisense _and RISC complexes. This also motivates the use of the Polyani equation for measuring the activation energy, which particularly works well for the following family of reactions:



Spectometric measurements on the thermodynamics of double-helix formation reported in Refs. [20] and [21] reveal the contribution of the 3' end in increasing the stability of the helix. Ref. [22] also relates the sequence dependence to the Gibb's free energy change to study the energetics of dangling ends and terminal base pairs in ribonucleic acid.

Hence, to measure the heat of reaction, we can use the heat of reaction required to dissociate the siRNA duplex. Note that, the second part of the reaction being essentially a docking reaction does not require any activation energy [23]. However, instead of using the actual heat of reaction (measured in terms of the change in enthalpy for the reaction), we plan to use the Gibb's free energy change (ΔG_37_^0^) that measures the "useful" or process-initiating work. The idea is that only the Gibb's free energy (which is a subset of the enthalpy or heat of reaction) is required for the overall reaction to occur, as the remaining heat will be generated by the docking part of the reaction. The docking reaction as mentioned before does not require any activation energy as it does not require changes in the chemical structure (i.e., no reaction is required), and in fact most binding events involve non-bonding interactions. As a consequence, such complexes may be formed by crossing small energy barriers that are within the range of thermal fluctuations (kT range at physiological temperature), and would not necessarily require a large kinetic energy during the collision. However, the electrostatic and solvation effects are the driving force for the formation of macromolecular complexes, and hence the docking part of the reaction. Hence, the activation energy, E_Act _will have the following components:

(12)

where, E_dissociation _is the energy required for the dissociation of the double-stranded siRNAs while E_electrostatic _and E_solvation_correspond to the electrostatic and solvation energy requirements for the docking reaction.

The Polyani equation for exothermic reactions is used for estimating E_dissociation _from the Gibb's free energy measurements as follows:

(13)

**Figure 4:**
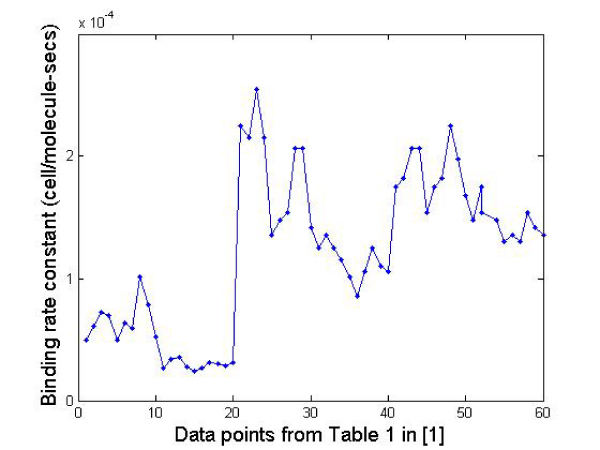
**Binding rate constant plot**.

A recent study on siRNA duplex stability [24] gives the thermodynamic energy requirements for 6-nt long siRNA duplex stability. We used the Gibb's free energy change measured by them for different types of residues present in the 2-nt overhangs to generate the binding rates predicted by our model. An 8-fold difference in the reaction rate was observed suggesting a purine residue followed by a purine/pyrimidine residue in the overhang results in an 8-fold slowdown of the reaction rate (the first 20 data points in Figure 4) compared to a pyrimidine residue followed by a purine/pyrimidine residue which supports the findings in Ref. [24]. The other parameters from the model (e.g., mass and radii of siRNAs and RISC enzymes) were arbitrarily assumed as we are interested in the *relative difference *in the binding rates for the different siRNAs instead of the actual values. Also, it should be noted that the mass and radii of the different siRNAs in Ref. [24] will be comparable and will not affect the binding rate appreciably.

**Figure 5:**
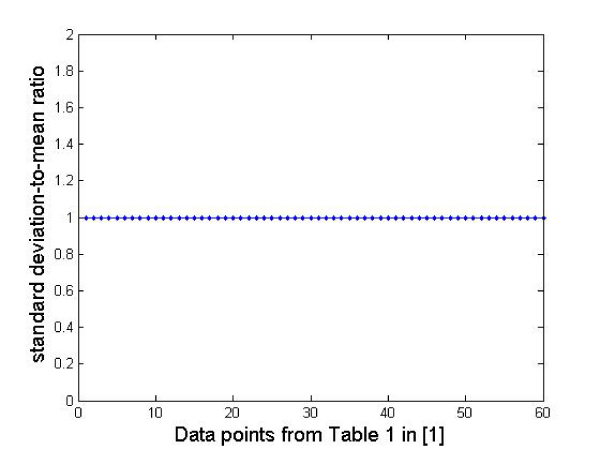
**The mean and standard deviation of reaction time are fairly equal and hence the reaction time is assumed to follow an exponential distribution**.

Ref. [24] also allows us to find out the distribution of the reaction time for the siRNA molecules reported from the Gibb's free energy estimates reported in Table 1 in Ref. [24]. In Figure 5, we plot the standard deviation-to-mean ratio of the reaction time from our model using the above-mentioned activation energy estimate. We observe that the standard deviation and mean times for reaction are the same, and hence the reaction time follows an exponential distribution. Thus, we can use the standard Gillespie stochastic simulator for studying the RNAi system where each reaction is considered stochastic, with the time for reaction completion following an exponential distribution.

**Table 1:** Simple RNAi system equations

	Reaction	Rate Constant
1	mRNA + siRNA -> gRNA	0.008 hr^-1^
2	dsRNA -> 10*siRNA	2.0 hr^-1^
3	mRNA -> dsRNA	0.002 hr^-1^
4	*ϕ*-> mRNA	160.0 hr^-1^
5	siRNA -> *ϕ*	2.0 hr^-1^
6	mRNA -> *ϕ*	0.14 hr^-1^
7	gRNA -> *ϕ*	2.8 hr^-1^

With the above findings, we estimated the Gibb's free energy change for dissociating the 21-nt siRNA duplexes of the following two types: 1) 21-nt siRNAs with 2-nt overhangs and 2) 21-nt blunt-ended siRNAs. The corresponding ΔG_37_^0 ^estimates are -21.2 kcal/mol and -20.4 kcal/mol respectively. The experimental setup is explained in the Appendix. This results in a 2-fold difference in the rate constant using these two types of molecules with the blunt-ended ones having a lower rate constant than their 2-nt overhang counterparts (i.e. T_avg_^blunt-end^/T_avg_^2-nt ^= 2, where T_avg_^blunt-end^and T_avg_^2-nt ^are the average reaction times for blunt-ended and 2-nt siRNAs respectively). Again we have assumed that the mass and radii of these two types of siRNAs are comparable and only the activation energy parameter from our reaction model makes the rate constant different. Also, note that the cell volume parameter cancels out as we only need to compute the ratio (T_avg_^blunt-end^/T_avg_^2-nt^) and not the actual rate constants. Similarly, we have assumed that E_electrostatic _and E_solvation _will be the comparable for the two types of siRNAs and will cancel out as we compute T_avg_^blunt-end^/T_avg_^2-nt^. Note that E_electrostatic _can be computed using the siRNA 3-d structures using standard software like Delphi [25] but will be very similar for the siRNAs that we have considered (which only differ by 2-nt at the 3' end). We next use this predicted difference in rate constants to compare the potency effects of these two types of molecules in an entire RNAi system simulation. Note that, because of the unavailability of some of the parameters required in the model (that we have assumed to be comparable for the two types of siRNAs), we have used the rate constant reported in Ref [26] for 2-nt siRNAs directly in the simulation model. To get the rate constant for blunt-ended siRNAs, we halved the rate constant as predicted by our reaction model.

### siRNA duplex stability measurements

Blunt-end and 2-nt 3' overhang siRNA's were reconstituted in buffer at 20 *μ*M. Stock solutions were then diluted in buffer to 10 *μ*M for data collection and determination of the thermodynamic parameters for siRNA unfolding. 120 *μ*L of solution was added to a micro-volume UV cuvette with a pathlength of 1.0 cm. The cuvette was placed in an AVIV UV/Vis spectrophotometer with a Peltier temperature controller. A reference cuvette filled with buffer solution was placed in the reference beam. The temperature was lowered to 10°C and the absorbance at 280 nm was auto zeroed. Temperature was then increased at 1°C/min and the change in absorbance recorded until the temperature reached 95°C. Absorbance data were exported to Meltwin 3.5 software [27] for data analysis. Data were fit to the self-complimentary model available in Meltwin 3.5.

## Results

### Stochastic simulation of the RNAi system

We primarily employed Dizzy [28] and the SimBiology toolkit from Matlab for the stochastic simulation of the RNAi system. Let us consider a simple RNAi system from Table 1. mRNA is transcribed according to reaction 4 and degraded according to reaction 6. dsRNA is synthesized from mRNA by RDR and is cleaved into 10 siRNAs according to reactions 3 and 2 respectively. siRNA can associate with mRNA according to reaction 1 to cleave the mRNA and produce garbage RNA (gRNA). For simplicity, we do not implement the formation of RISC explicitly in our model; instead, the siRNA-mRNA complex is directly degraded into aberrant gRNA. Thus, we assume that the RISC enzymes are not a rate limiting component of the RNAi system. Reactions 5 and 7 describe the degradation of siRNAs and aberrant garbage pieces, respectively. This simple RNAi system is motivated from [26] from where we get an estimate of the basic rate constants. We did not consider any nonspecific effects following the observation in Ref. [29] that siRNA-mediated inhibition of gene expression is generally independent of nonspecific interference pathways triggered by the dsRNAs. Our goal in this study is to present a systems biology framework for studying the potency effects of the two types of siRNA based on the difference in their rate constants. Thus, the rate constant of reaction 1 is 0.008 for siRNAs with 2-nt overhangs while it will be approximately 0.004 for the blunt-ended molecules.

**Figure 6:**
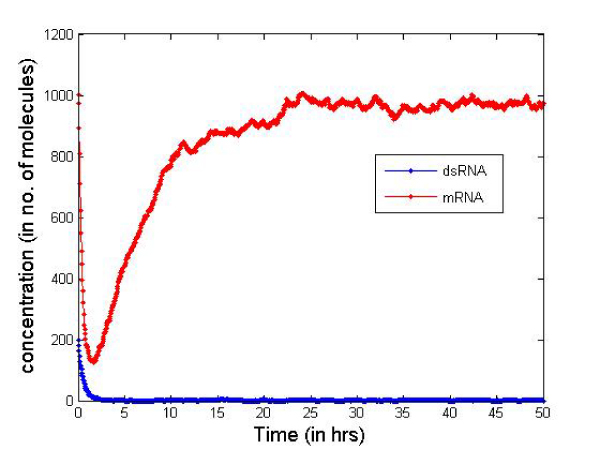
**Concentration change using 2-nt 3'siRNAs**.

**Figure 7:**
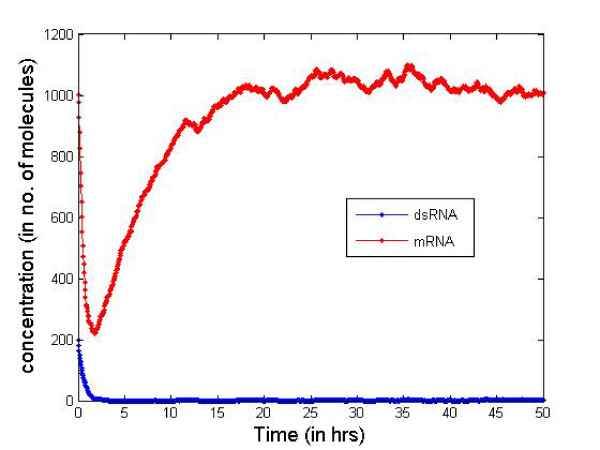
**Concentration change blunt-ended siRNAs**.

**Figure 8:**
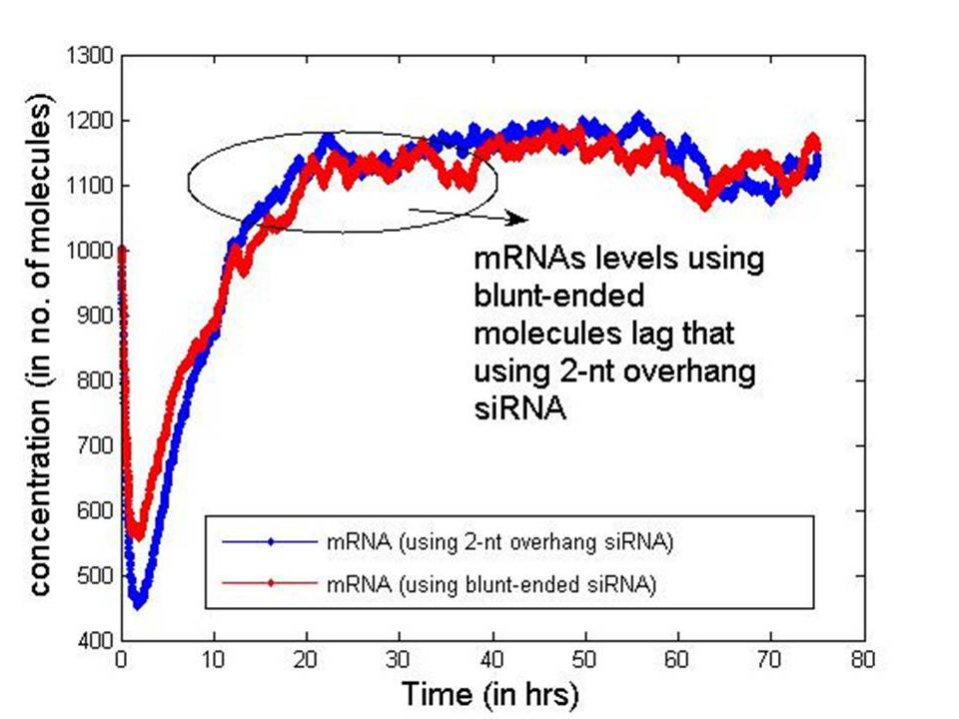
**mRNA levels saturate after longer time while blunt-ended siRNAs are used**.

Table 2 presents the initial number of molecules considered in the cell for the simulation. These values are just for illustrative purposes but serve well to compare the two types of siRNAs. In Figures 6 and 7 we report the change in concentration of the dsRNA and mRNA molecules with time (in hours) for the 2-nt overhangs and blunt-ended molecules respectively. Note that the dsRNA molecules get used up quite quickly (in less than 3 hours) in both cases depending on the siRNA production rate from dsRNAs and dsRNA degradation. However, for blunt-ended siRNAs, the mRNA levels go down to about 250 molecules (in about 3 hours) compared to about 150 molecules when the 2-nt overhang siRNAs are used. This is obvious as the siRNA-RISC complex formation reaction rate for blunt ended molecules is about half of that for 2-nt overhang siRNAs. Another point worth noting is that the mRNA levels return to control levels or untreated levels after a longer period when blunt-ended molecules are used. This can be attributed to the fact that the rate constant for the siRNA-RISC complex formation reaction being low for blunt-ended molecules, the blunt-ended siRNAs persist in the system for a longer time than the 2-nt 3' overhang counterparts. Figure 8 illustrates this phenomenon, where the mRNA levels using blunt-ended siRNAs seem to lag behind that observed for the 2-nt overhang species. The plot was generated with the initial concentration of dsRNA molecules set to 100 for illustrative purposes.

**Table 2:** Initial conditions

Reactant	Number of molecules
mRNA	1000
siRNA	0
dsRNA	200
gRNA	0

Next we consider the transcription/translation machinery to study the potential effects of stochastic mRNA production on the RNAi system [30]. The additional reactions (7–18) were added to consider the transcription/translation processes (presented in Table 3) and the initial conditions are reported in Table 4. A single gene is transcribed into mRNA by RNA polymerase (RNAP). The process is initiated with the binding of RNAP to the promoter, usually near the beginning of the transcribed sequence. Expression of most genes are regulated at the level of transcription and more specifically during the initiation of transcription, that is, before the first phosphodiester bond is formed.

**Table 3:** RNAi system with transcription/translation machinery

Reaction Number	Reaction	Rate Constant	Remarks
1	mRNA + siRNA -> gRNA	0.008 hr^-1^	
2	dsRNA -> 10*siRNA	2.0 hr^-1^	
3	mRNA -> dsRNA	0.002 hr^-1^	
4	siRNA -> *ϕ*	2.0 hr^-1^	
5	mRNA -> *ϕ*	18 hr^-1^	
6	gRNA -> *ϕ*	2.8 hr^-1^	
7	O + R -> O_R	12.0 hr^-1^	Regulatory molecule R binds to the operator region O to form the bound complex O_R
8	O_R -> O + R	0.24 hr^-1^	O_R dissociates into free R and O
9	P + RNAP -> P_RNAPC	1.2 hr^-1^	RNAP binds to promoter region P forming closed complex P_RNAPC
10	P_RNAPC -> P + RNAP	0.06 hr^-1^	P_RNAPC dissociates into free RNAP and P
11	P_RNAPC -> P_RNAPO	48 hr^-1^	Isomerization of closed to open complex (P_RNAPO)
12	P_RNAPO -> TrRNAP + mRNA + P	54 hr^-1^	RNAP clears promoter region and mRNA chain synthesis starts. TrRNAP denotes transcribing RNA polymerase.
13	TrRNAP -> RNAP	4.8 hr^-1^	RNAP completes transcription and is released from DNA.
14	mRNA + Ribosome -> RibRBS	0.6 hr^-1^	Ribosome binds to mRNA forming bound complex RibRBS
15	RibRBS -> mRNA+ Ribosome	0.06 hr^-1^	Ribosome dissociation from RibRBS
16	RibRBS -> EIRib + mRNA	18 hr^-1^	Ribosome EIRib initiates translation of mRNA chain
17	EIRib -> protein	48 hr^-1^	Protein synthesis by transcribing ribosome
18	protein -> *ϕ*	0.18 hr^-1^	Protein product degradation

**Table 4:** Initial Conditions for the combined RNAi system

Reactant	Number of molecules
mRNA	1000
siRNA	0
dsRNA	200
gRNA	0
R	20
O	1
O_R	0
P	200
RNAP	400
P_RNAPC	0
P_RNAPO	0
TrRNAP	0
Ribosome	350
RibRBS	0
EIRib	0
protein	0

**Figure 9:**
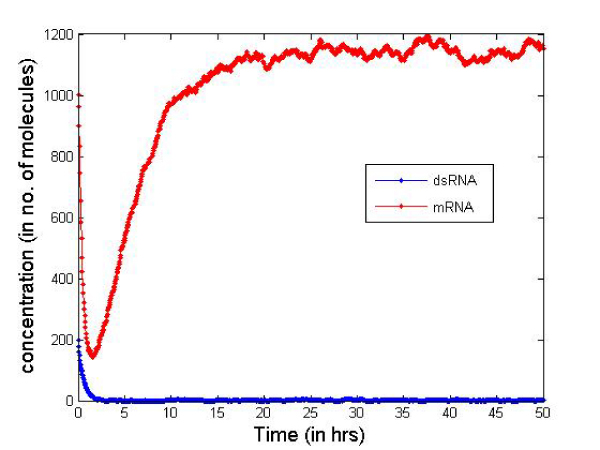
**Combined RNAi system plot using 2-nt 3'siRNAs**.

**Figure 10:**
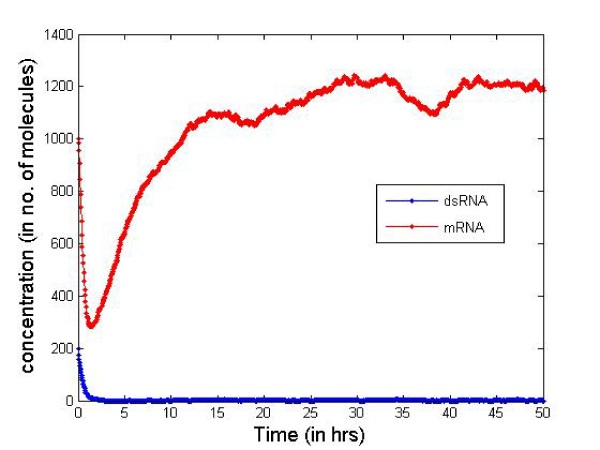
**Combined RNAi system plot using blunt-ended siRNAs**.

**Figure 11:**
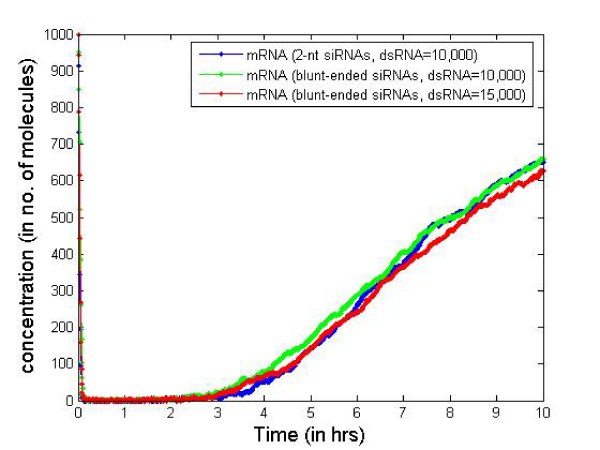
**Quantification of gene silencing period with different types of siRNAs**.

Figures 9 and 10 report the mRNA and dsRNA concentration changes with time for this combined RNAi system. We observe similar effects (both quantitative and qualitative) as in the simple system without considering the transcription/translation reactions. Note that the simple system considered a certain mRNA production rate and does not capture the uneven mRNA production observed recently [16]. Because of stochastic gene expression in eukaryotic cells, mRNA and hence protein production is erratic and occurs in bursts. The average intervals between bursts is longer in eukaryotes compared to prokaryotes [16]. Thus, the stochasticity in gene expression does not have any observable effects in the RNAi system. One plausible reason behind this is the fact that the stochasticity is observed at a much lower time scale (generally in seconds) while we in this simulation, are interested in studying the mRNA concentrations over a longer time scale (hours). Initial findings show about 1.5 times more blunt-ended molecules will be required to keep the mRNA at a certain low level. Figure 11 plots the mRNA levels for different types of siRNAs in order to quantify the gene silencing period. With 10,000 dsRNA molecules for the two types of siRNAs, the silenced period is larger for 2-nt overhang siRNAs. Using 15,000 dsRNAs of blunt-ended type we can achieve the same amount of silencing as the 2-nt overhang siRNAs.

**Figure 12:**
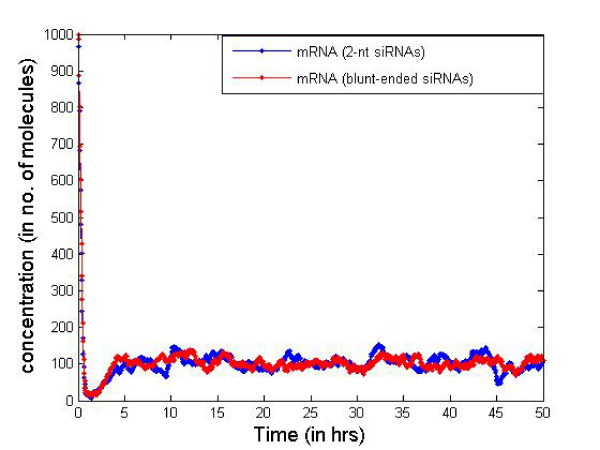
**2-nt 3' and blunt-ended siRNAs with RDR show the mRNA/dsRNA concentration changes with time at low amplification through RDR for the two types of siRNAs considered**. We do find an expected increase in the potency effects for both types of siRNAs (compared to Figures 6 and 7).

**Figure 13:**
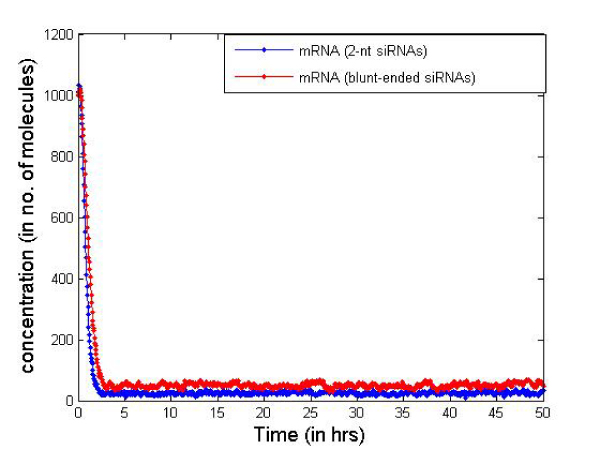
**2-nt 3' and blunt-ended siRNAs with RDR**. Figure 13 shows the effects of high amplification through RDR and we can observe *sustained gene silencing*. The actual mRNA levels will however depend on the amplification rates and initial number of dsRNA molecules inserted into the cell.

**Figure 14:**
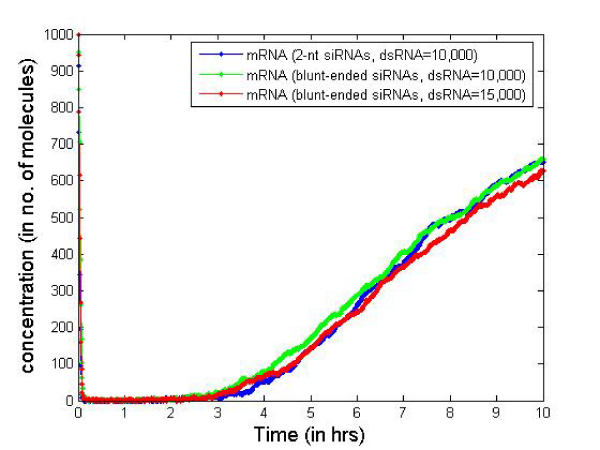
**2-nt 3'and blunt-ended siRNAs with artificial dsRNA injection**. Fiureg 14 show the effects of artificial dsRNA injection (for the simple RNAi system of Table 1) for the two types of siRNAs with 200 dsRNA molecules inserted every hour into the cell.

Next consider the effects of the RDR mechanism of replenishing the dsRNAs in the cell. Using the simple RNAi system (of Table 1), as the transcription/translation machinery failed to show any significant changes in the system behavior. Table 5 lists the additional reactions to incorporate the RDR mechanism. Reaction 8 describes the unprimed amplification – the synthesis of dsRNA from aberrant garbage RNA by RDR; and reaction 9 describes primed amplification – the synthesis of dsRNA primed by the presence of a siRNA on mRNA. We consider the pathway with the amplification terms at both low and high amplification rates (Figures 12 and 13 respectively). Thus sustained gene silencing can only occur through the RNAi pathway if there is a way of replenishing the dsRNAs in the cell. This can occur through the RDR mechanism (with high amplification as illustrated in Figure 13), or through artificial dsRNA injection into the cell (Figure 14) at certain time periods. There is no conclusive evidence of the presence of the RDR mechanism in mammalian cells, and hence the artifical dsRNA injection will play a very important role in any RNAi based therapeutic tool.

**Table 5:** Additional reactions from Table 1 to consider the RDR mechanism

Reaction Number	Additional Reactions from Table 2	High Amplification	Low Amplification
8	gRNA -> dsRNA	0.4 hr^-1^	0.02 hr^-1^
9	mRNA + siRNA -> dsRNA	0.002 hr^-1^	0.0002 hr^-1^

There is a slight difference in the mRNA levels with the 2-nt overhang siRNAs fairing a little better than their blunt-ended counterparts. However, we can get a relative quantification of the number of dsRNA molecules required to be inserted into the cell for two types of siRNAs considered to keep the mRNAs down at the same level. This will require more real-life initial concentration values for the other components of the RNAi system and also a model to predict the average number of dsRNA molecules that actually enter a single cell depending on the dosage amount and intervals. Our model provides a simulation framework that will help us study the gene silencing duration using siRNAs in the future.

## Discussion

### Handling delayed reactions

We have computed the time taken to complete the reaction. The underlying assumption is that reactant collisions occur with some probability and once a collision of sufficient energy occurs, a reaction takes place instantaneously. Hence, we assume that there is no time delay to form an activated complex. If there is some time delay associated with initiation and completion of the reaction, the probability evolution becomes more complicated [31]. Our reaction model cannot directly handle such delayed reactions, which would require comprehensive modeling of the delayed states. However, the reaction model in this paper was primarily developed for the siRNA-RISC complex formation reaction, which does not require the handling of delayed reactions. However, this involves an implicit approximation that the reactant molecules are not available for other reactions once it has entered the present reaction event. But because both the original reaction event time and the delay can be random variables incorporating the probability of successfully completing the delayed reaction event, this approximation should be small. Further analysis is required to study the effect of this approximation.

### Maxwell-Boltzmann distribution of molecular velocities

The Maxwell-Boltzmann distribution gives a good estimate of molecular velocities where we have spatial homogeneity and is widely used in practice. Molecular dynamic (MD) simulation measurements during protein reactions show that the velocity distribution of proteins in the cytoplasm closely matched the Maxwell-Boltzmann distribution [18,19]. However, its application in our model may not give the predicted results for cases that violate the assumption of uniform distribution in a volume. Ideally the velocity distribution should incorporate the properties of the reaction space (nucleus/membrane/cytoplasm for reactions occurring in the membrane, nucleus or cytoplasm) and the effect on velocity distribution due to its space shape and irregularities. We plan to explore the possibility to improve this velocity distribution by considering the other biological factors that can influence the velocity of the reacting molecules.

### Activation energy threshold

The activation energy has been measured for many reactions and we need an estimate of this parameter to be able to predict the nature of the reaction time. We used the Polyani equation to compute E_dissociation _for the reaction by measuring the Gibb's free energy change to dissociate a siRNA duplex. As discussed before, this approximation can give us a relative difference in the reaction rates for the two types of siRNAs used in our study, but will fail to give us a direct quantification of the actual reaction rates (which will also require estimates of E_electrostatic_, E_solvation_, mass, radii etc. for the siRNAs). We are exploring ways to measure the actual heat of reaction for the entire siRNA-RISC complex formation reaction and the other parameters to make the model predictions more accurate.

### Reverse reactions

We did not consider the reverse reaction conditions in our model because we assumed the siRNA-RISC complex formation reaction as non-reversible. However, the Gillespie stochastic simulation framework allows us to incorporate reversible reactions if we know the forward and backward reaction rates [15]. We plan to make the simulation framework presented in this paper more comprehensive once we have experimental measure of these reaction rates.

### Reaction neighborhoods

In addition, there is increasing evidence of sub-compartmental (i.e., intra-compartmental localization) in cells, so local neighborhoods of reactions will have higher apparent concentrations than simply the number of molecules divided by the size of the compartment. However, this would require more in depth modeling of the different molecular concentrations inside the cell that reduces the scalability of the stochastic simulation framework. Indeed, the Gillespie simulator fails to address this issue as it requires different rate constants for specific neighborhoods of the reaction type. Nevertheless, our model can be easily extended to incorporate such reaction neighborhoods by limiting the movement of the reactant molecules inside a reaction space while computing the probability. However, the applicability of the Maxwell-Boltzmann velocity distribution in the neighborhood requires further research.

### Role of GW/P-bodies in RNA silencing

A related concern with this simulation model is the recent finding that RISC and other enzymes involved in RNA degradation tend to localize to discrete cytoplasmic foci known as P-bodies [32,33]. Moreover, siRNAs were also observed to localize to specific cytoplasmic compartments in the periphery of the nucleus in granular-like structures [34]. This compartmentalization will have an effect on the reaction model that we have developed based on the entire cell volume. However, it should be noted that the reaction model is only used to find the relative difference in the reaction rates between the two types of siRNAs and not to predict the overall reaction rate. We have used the reaction rates reported in Ref [26] in our simulation model and changed it accordingly for blunt ended siRNAs using the reaction model. Also, finding the relative difference in the reaction rates would mean the cancellation of the parameter *V*, and hence is independent of the effects of this compartmentalization. It can be argued that the simulation model however requires additional reactions to address the effect of GW/P-bodies (e.g., by adding additional delay for siRNAs to reach the cytoplasmic foci and additional reactions for forming the complex with GW/P-bodies). This will require additional research to estimate the kinetic parameters for these reactions. In this paper, however, we can neglect the effect of GW/P-body formations because the reaction rate mentioned in Ref [26] is for the overall siRNA-mRNA complex formation reaction and should already incorporate the effects of this compartmentalization.

### 27-mer RNA duplexes show higher potency than 21-mer siRNAs

Many researchers today employ synthetic 21 mer RNA duplexes as their RNAi reagents, which mimic the natural siRNAs that result from Dicer processing of dsRNAs. An alternative approach is to use synthetic RNA duplexes that are greater than 21 mer length which are substrates for Dicer. These duplexes are typically 27-nt long and are processed by Dicer into 21 mer siRNAs. It has been reported that synthetic Dicer-substrate RNAs can have significantly increased potency (~100-fold) when compared with 21 mer duplexes [35,36]. The 100-fold increase in potency of the 27 mer is due to a combination of Dicer cleavage resulting in "a better 21 mer" (10-fold increase) plus some other effect that required use of the intact 27 mer (another 10-fold increase).

More recent works targeted additional sites in other genes, to find examples where the longer RNAs had greater potency, the same potency, and lower potency than 21 mers at the same site. This wide variation in performance was primarily attributed to the differences in dicing patterns: sometimes Dicer processing resulted in a "good" 21 mer while other times it resulted in a "bad" 21 mer (Ref [14]). Small shifts in sequence can have a large effect on siRNA potency. Combination of asymmetric 2-base 3'-overhang with 3'-DNA residues on the blunt end result in a duplex form which directs dicing to predictably yield a single primary cleavage product. Using this strategy, strand targeting experiments in Ref [14] show that Dicer processing confers functional polarity within the RNAi pathway.

In this work, we have not studied the effects on potency while using synthetic 27 mers instead of the dsRNAs. This will require the ratios of the "good" and "bad" 21 mers that are diced from the 27 mers entering the cell as well as a similar thermodynamic stability study of these 21 mers to study their effects on the rate constant estimates.

## Conclusion

The RNAi system simulation framework developed here presents some qualitative and quantitative results on the RNAi pathway. The simulation also explores the potency effects of the two types of siRNAs considered. We primarily focused on identifying the most important components of the system that play a role in identifying the potency effects. This framework can be extended to predict very important features of the RNAi pathway including the duration of gene silencing for the different siRNA molecules. This will require models to study the effective rate of dsRNA entry into the cell depending on the amount of dosage and dosage intervals. We plan to study these important aspects of the RNAi system, once we build more fidelity on this simulation framework.

The proposed model computes the reaction time for the siRNA-RISC complex formation reaction as a stochastic variable that appropriately reflects the cell environment. The concept of the model is to transform the reaction process from a continuous deterministic process to a discrete random one. The model allows the transformation of biological reactions to the stochastic domain and makes it suitable for a stochastic simulation of the RNAi system. The average reaction time estimated from this method is exactly the same as the reaction rate estimates of kinetic modeling. In addition, we are able to estimate the first two moments of the reaction time to capture the stochastic nature of the reaction. The reaction model was used to study the difference in binding rate for the 2-nt 3' overhang siRNAs and the blunt-ended siRNAs, and we found that the reaction rate is predicted to double when 2-nt 3' overhang siRNAs are used. We next built an RNAi stochastic system simulation using the Gillespie simulator to study the overall potency effects of the two types of siRNAs. Initial findings suggest that about 1.5 times more blunt-ended siRNAs are required to keep down the mRNA at the same level as using 2-nt 3' overhang siRNAs. The additional blunt-end siRNAs may be needed because the siRNA-RISC complex formation reaction is not the only part of the RNAi pathway that is affected by the presence of the 2-nt overhangs in the siRNA molecules. The reaction in which the bound RISC complex cleaves the mRNA might be affected more due to the difference in the siRNA structures. Further research is required to study this aspect of the RNAi system.

The stochastic simulation framework presented here allows us to predict some quantitative and qualitative aspects of the RNAi system for the two different types of siRNAs used in our study. More importantly, it allows us to build a quantitative framework for studying the length of the gene silencing period and number of siRNA molecules (of both types) required for the same for a cost-potency study. This will require a model to estimate the average number of dsRNA molecules actually entering the cell depending on the dosage interval and amount. The initial predictions from our work are promising, as we plan to build a computational tool for studying the therapeutic effects of the RNAi pathway.

## Competing interests

The authors declare that they have no competing interests.

## Authors' contributions

All authors designed, analyzed, implemented and tested the proposed models. Each author contributed equally in writing the paper. All authors read and approved the final manuscript.

## References

[B1] Milhavet O, Gary DS, Mattson MP (2003). RNA interference in biology and medicine. Pharmacol Rev.

[B2] Tuschl T, Zamore PD, Lehmann R, Bartel DP, Sharp PA (1999). "Targeted mRNA degradation by double-stranded RNA in vitro". Genes Dev.

[B3] Elbashir SM, Harborth J, Lendeckel W, Yalcin A, Weber K, Tuschl T (2001). Duplexes of 21-nucleotide RNAs mediate RNA interference in cultured mammalian cells. Nature.

[B4] McManus MT, Sharp PA (2002). Gene silencing in mammals by small interfering RNAs. Nat Rev Genet.

[B5] Miyagishi M, Hayashi M, Taira K (2003). Comparison of the suppressive effects of antisense oligonucleotides and siRNAs directed against the same targets in mammalian cells. Antisense Nucleic Acid Drug Dev.

[B6] Reynolds A, Leake D, Boese Q, Scaringe S, Marshall WS, Khvorova A (2004). Rational siRNA design for RNA interference. Nat Biotechnol.

[B7] Yoshinari K, Miyagishi M, Taira K (2004). Effects on RNAi of the tight structure, sequence and position of the targeted region. Nucleic Acids Res.

[B8] Elbashir SM, Lendeckel W, Tuschl T (2001). RNA interference is mediated by 21 and 22 nt RNAs. Genes Dev.

[B9] Zamore PD, Tuschl T, Sharp PA, Bartel DP (2000). "RNAi: Double-stranded RNA directs the ATP-dependent cleavage of mRNA at 21 to 23 nucleotide intervals". Cell.

[B10] Sijen T, Fleenor J, Simmer F, Thijssen KL, Parrish S (2001). On the role of RNA amplification in dsRNA-triggered gene silencing. Cell.

[B11] Lipardi C, Wei Q, Paterson BM (2001). RNAi as random degradative PCR: siRNA primers convert mRNA into dsRNAs that are degraded to generate new siRNAs. Cell.

[B12] Makeyev EV, Bamford DH (2002). Cellular RNA-dependent RNA polymerase involved in posttranscriptional gene silencing has two distinct activity modes. Mol Cell.

[B13] Schiebel W, Haas B, Marinkovic S, Klanner A, Sanger HL (1993). RNA-directed RNA polymerase from tomato leaves. II. Catalytic in vitro properties. J Biol Chem.

[B14] Rose SD, Kim DH, Amarzguioui M, Heidel JD, Collingwood MA, Davis ME, Rossi JJ, Behlke MA (2005). Functional polarity is introduced by Dicer processing of short substrate RNAs. Nucleic Acids Res.

[B15] Gillespie D Exact stochastic simulation of coupled chemical reactions. Journal of Physical Chemistry.

[B16] MacAdams H, Arkin A (1999). It is a noisy business! Genetic regulation at the nanomolar scale. Trends in Genetics.

[B17] Masel RI (2001).

[B18] Lin DTW, Yang C (2005). The heat transfer analysis of nanoparticle heat source in alanine tissue by molecular dynamics. International Journal of Biological Macromolecules.

[B19] Fogler H, Gurmen M Elements of Chemical Reaction Engineering Chapter 31, Equation 13, online at.

[B20] Petersheim M, Turner DH (1983). Base-stacking and base-pairing contributions to helix stability: thermodynamics of double-helix formation with CCGG, CCGGp, CCGGAp, ACCGGp, CCGGUp, and ACCGGUp. Biochemistry.

[B21] Freier SM, Burger BJ, Alkema D, Neilson T, Turner DH (1983). Effects of 3' dangling end stacking on the stability of GGCC and CCGG double helicies. Biochemistry.

[B22] Sugimoto N, Kierzek R, Turner DH (1987). Sequence dependence for the energetics of dangling ends and terminal base pairs in ribonucleic acid. Biochemistry.

[B23] Ghosh P, Ghosh S, Basu K, Das S (2007). "A Computationally Fast and Parametric Model to Estimate Protein-Ligand Docking Time for Stochastic Event Based Simulation". Lecture Notes in Computer Science.

[B24] O'Toole AS, Miller S, Haines N, Zink MC, Serra MJ (2006). "Comprehensive thermodynamic analysis of 3' double-nucleotide overhangs neighboring Watson-Crick terminal base pairs". Nucleic Acids Research.

[B25] Oron A, Wolfson H, Gunasekaran K, Nussinov R (2003). Using DelPhi to compute electrostatic potentials and assess their contribution to interactions. Curr Protoc Bioinformatics.

[B26] Groenenboom MAC, Maree AFM, Hogeweg P (2005). "The RNA Silencing Pathway: The Bits and Pieces That Matter". PLOS Computational Biology.

[B27] Meltwin v3.5 Copyright 1996, 1997 by Jeffrey A. McDowell 36147 N. Tangueray Dr. Grayslake, IL 60031 USA All Rights Reserved.

[B28] Dizzy, Institute for Systems Biology-CompBio Group. http://magnet.systemsbiology.net/software/Dizzy/.

[B29] Caplen NJ, Parrish S, Imani F, Fire A, Morgan RA (2001). Specific inhibition of gene expression by small double-stranded RNAs in invertebrate and vertebrate systems. Proc Natl Acad Sci USA.

[B30] Bose I, Karmakar R, Roy S (2002). "Stochastic Simulation of Gene Expression in a Single Cell". ArXiv Condensed Matter e-prints.

[B31] Bratsun D, Volfson D, Tsimring L, Hasty J (2005). Delay-induced stochastic oscillations in gene regulation. Proceedings of the National Academy of Sciences.

[B32] Liu J, Valencia-Sanchez MA, Hannon GJ, Parker R (2005). MicroRNA-dependent localization of targeted mRNAs to mammalian P-bodies. Nat Cell Biol.

[B33] Sen GL, Blau HM (2005). Argonaute 2/RISC resides in sites of mammalian mRNA decay known as cytoplasmic bodies. Nat Cell Biol.

[B34] Lavigne C, Thierry AR (2007). Specific subcellular localization of siRNAs delivered by lipoplex in MCF-7 breast cancer cells. Biochimie.

[B35] Meister G, Tuschl T (2004). Mechanisms of gene silencing by double-stranded RNA. Nature.

[B36] Kim DH, Behlke MA, Rose SD, Chang MS, Choi S, Rossi JJ (2005). Synthetic dsRNA Dicer substrates enhance RNAi potency and efficacy. Nat Biotechnol.

